# Chronic hepatitis E infection with an emerging virus strain in a heart transplant recipient successfully treated with ribavirin: a case report

**DOI:** 10.1186/s13256-015-0655-z

**Published:** 2015-08-26

**Authors:** Jesper Waldenström, Maria Castedal, Jan Konar, Kristjan Karason, Martin Lagging, Helene Norder

**Affiliations:** 1 Department of Infectious Diseases/Virology, Institute of Biomedicine, Sahlgrenska Academy, University of Gothenburg, Gothenburg, Sweden; 2 Transplant Institute, Sahlgrenska University Hospital, Sahlgrenska Academy, University of Gothenburg, Gothenburg, Sweden; 3 Department of Transfusion Medicine, Sahlgrenska Academy, University of Gothenburg, Gothenburg, Sweden; 4 Institute of Medicine, Sahlgrenska Academy, University of Gothenburg, Gothenburg, Sweden

**Keywords:** Chronic, Genotype 3, Heart transplant, Hepatitis E virus, HEV, Ribavirin, Transfusion transmitted

## Abstract

**Introduction:**

During the last decade hepatitis E infections have been recognized as a health problem in high-income countries, where hepatitis E virus genotype 3 is endemic. The infection is often self-limiting, but may develop into chronic infection in immunocompromised patients, especially in solid organ recipients. If these patients or patients with underlying liver disease get hepatitis E infection, they may develop liver failure and cirrhosis. Hepatitis E virus is occasionally found in blood products and transfusion transmission has been reported. We present the first case of chronic hepatitis E infection in a heart transplant recipient in Sweden.

**Case presentation:**

A 63-year-old Swedish white man presented with highly elevated liver enzymes 6 months after heart transplantation. Polymerase chain reaction revealed chronic hepatitis E infection, caused by a virus strain found infecting symptomatic cases in Sweden and other European countries. During transplantation, he received blood products from 17 donors, and transfusion transmission is highly likely. The only detectable marker for hepatitis E infection was hepatitis E virus ribonucleic acid for more than 2 months before anti-hepatitis E virus developed. He was treated successfully with ribavirin and decreased immunosuppression.

**Conclusions:**

Our patient was probably infected through contaminated blood products and subsequently developed chronic infection, which was cured upon treatment. This highlights the need for evaluating the problem with chronic hepatitis E infection in immunocompromised patients, and for discussion concerning screening of blood products. Polymerase chain reaction-based methods are recommended for diagnosing hepatitis E infection in patients with compromised immunity. In addition, knowledge needs to be gained on the infecting virus strain, which may be more virulent than other strains.

## Introduction

Hepatitis E virus (HEV) belongs to the *Hepeviridae* family and is a non-enveloped positive-sense single-stranded ribonucleic acid (RNA) virus with a 7.2kb genome encoding three open reading frames (ORF 1 to 3). There are at least four different genotypes (gt) of HEV infecting humans (gt 1 to gt 4). During the last decade, HEV, particularly gt 3, has been found to be endemic in many developed countries and seroprevalence studies suggest that the infection is more common than previously believed [1]. The transmission routes of gt 3 infections are not fully understood, but the most common is probably zoonotic: fecal–oral transmission via undercooked meat or direct contact with infected pigs or other animals [2]. Transmission by transfusion of blood products has also been reported [3–7], as routine screening of blood products is not currently implemented.

The infection with HEV gt 3 is often self-limiting and asymptomatic in immunocompetent individuals, but patients with underlying liver disease may develop liver failure [2, 8, 9]. The incubation period is usually 2 to 6 weeks [10], during which HEV RNA can be detected in sera and stool just prior to onset of symptoms. The viremia persists for approximately 3 weeks, and HEV RNA can be detected in stool for an additional 2 weeks during acute self-limiting infection [11]. Both anti-HEV immunoglobulin (Ig) M and IgG can usually be detected shortly after viremia develops [12]. However, serologic analysis is not reliable in immunocompromised patients, in whom the antibody response is often delayed or undetectable. Such patients are at risk of becoming chronic carriers [13–15]. In solid organ recipients the risk of developing chronicity is reported to be as high as 60% and chronic hepatitis may lead to rapid development of cirrhosis [16, 17].

This study presents a case of chronic hepatitis E infection (HE) in a patient who underwent heart transplantation 21 days before HEV RNA was detected in his serum. The route of transmission remains unknown, although administration of contaminated blood products appears most likely. The infecting strain was phylogenetically analyzed and compared to strains infecting individuals with symptomatic HEV infection.

## Case presentation

A 63-year-old Swedish white man underwent orthotopic heart transplantation in July 2012 secondary to dilated cardiomyopathy. Preoperative liver enzymes and bilirubin were within normal ranges with the exception of a slightly elevated aspartate aminotransferase (AST; 1.3 times the upper limit of normal). He had an elevated prothrombin time-international normalized ratio (PT-INR) consistent with his warfarin treatment for atrial fibrillation. Virus serology was negative for hepatitis C virus and hepatitis B virus and he was immune against hepatitis A virus. In addition, he had antibodies against cytomegalovirus (CMV) and Epstein–Barr virus.

The surgery was complicated by excessive bleeding, which required administration of clotting factors, plasma and thrombocytes. Postoperatively, he developed a moderate worsening of a pre-existing renal insufficiency and thrombocytopenia. Also, a transient rise in bilirubin and liver enzymes was noted. The maintenance immunosuppressive therapy consisted of tacrolimus (target 6 to 8μg/L), everolimus (target 3 to 6μg/L), mycophenolate mofetil 1g twice a day and prednisolone 15mg daily. One month after surgery he was discharged with normal transaminases.

### Diagnosis

**Fig. 1:**
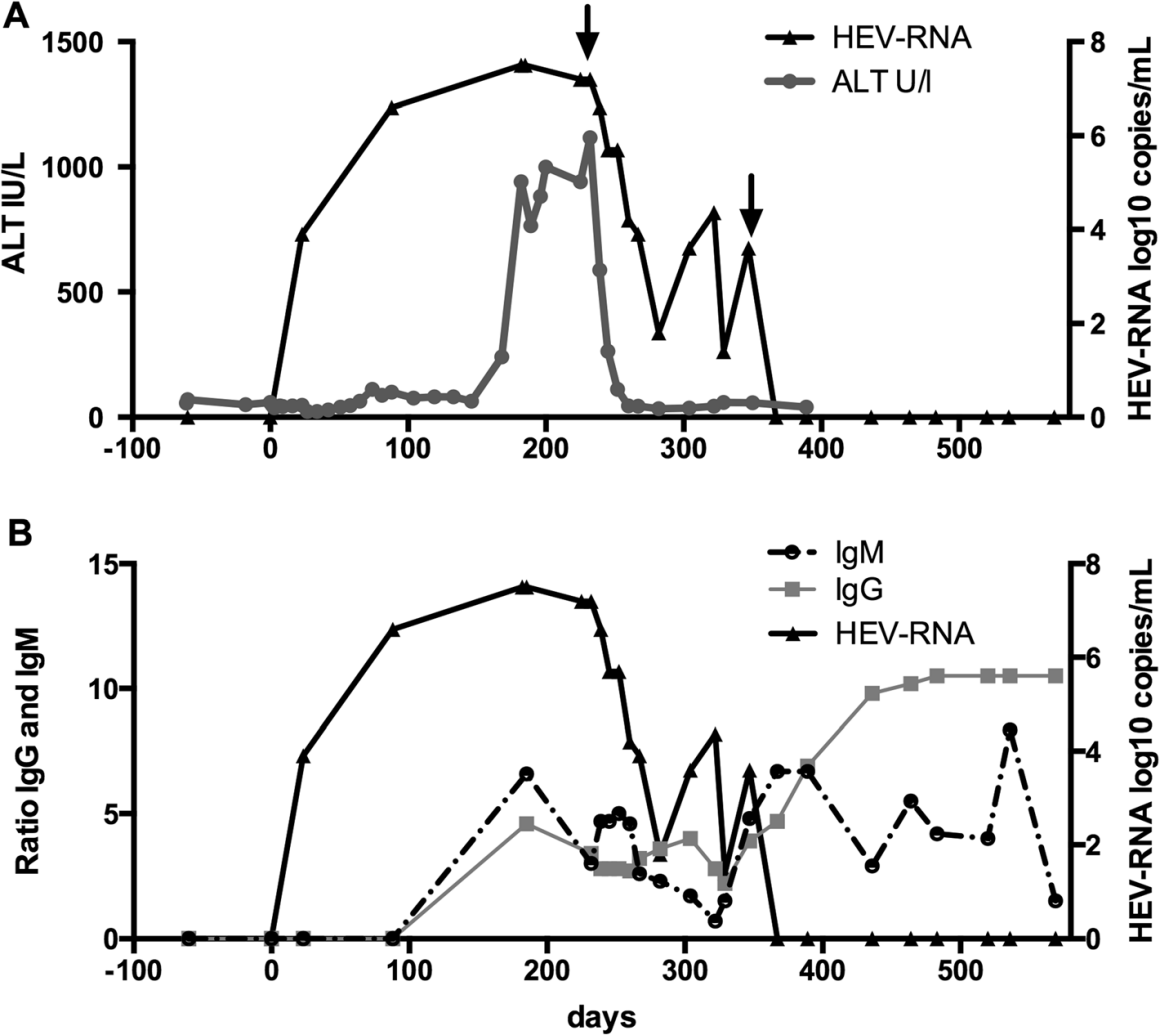
**a** Liver enzymes, copies of hepatitis E virus ribonucleic acid and **b** antibodies against hepatitis E virus are shown in days post-heart transplantation. *Arrows* indicate initiation of ribavirin treatment and dose increase. Alanine aminotransferase (*ALT*; IU/mL), hepatitis E virus (*HEV*) ribonucleic acid (*RNA*; log10/mL), immunoglobulin G (*IgG*) anti-hepatitis E virus threshold 1.0 and immunoglobulin M (*IgM*) anti-hepatitis E virus threshold 1.0

At 6-month follow-up, a marked elevation of liver enzymes was noted and, retrospectively, a borderline enzyme elevation was observed 2 months post-surgery (Fig. 1a). An abdominal ultrasound showed a suspected liver hemangioma, but was otherwise normal. Echocardiography revealed moderate pericardial effusion without hemodynamic compromise, and normal cardiac function. An endomyocardial biopsy showed no signs of significant rejection. Drug-induced hepatotoxicity was initially suspected because of a temporally related increase in simvastatin dosing, but discontinuation of this drug had no effect on his liver enzymes. Autoantibodies, antinuclear antibodies (ANA), smooth muscle antibodies (SMA) and antimitochondrial antibodies (AMA) for autoimmune hepatitis were all negative. He had not seroconverted in markers for hepatitis B or C infection. He had a low titer of CMV DNA in sera consistent with minor reactivation, which was absent at follow-up a week later.

Serology for HEV was reactive for both anti-HEV-IgM and IgG-antibodies (recomWell HEV IgG/IgM enzyme-linked immunosorbent assay, Mikrogen Diagnostik, Germany), and HEV RNA (1.6×10^7^ copies per mL) was detected in serum by quantitative polymerase chain reaction (PCR) [18] at the 6-month follow-up. A retrospective investigation revealed that the patient was negative for anti-HEV and HEV RNA 2 months prior to surgery and at the day of surgery, but HEV RNA was detected 3 weeks post-surgery in the first available serum sample post-transplantation (Fig. 1a, b). At that time point, the serology for HEV was negative and remained negative for the next 9 weeks despite detectable HEV RNA. Both anti-HEV IgM and IgG were detectable in the next available serum sample, which was obtained 26 weeks post-surgery (Fig. 1b).

### Treatment

Six months after surgery, the immunosuppressive therapy had been reduced, and consisted of tacrolimus (target 3 to 6μg/L), everolimus, (target 3 to 6μg/L), mycophenolate mofetil 0.5g twice a day, and prednisolone 7.5mg daily. After diagnosis of HEV infection, the dose of mycophenolate mofetil was reduced further to 0.25g twice a day and treatment with ribavirin initiated at a dosing of 800mg daily. Ribavirin therapy resulted in a rapid decline of liver enzymes and after 2 months of treatment, the level of HEV RNA in his serum dropped from 1.6×10^7^ to 63 copies per mL (Fig. 1). He then experienced an exacerbation of gout, which was treated by increasing prednisolone dosing to 20mg daily for 3 days followed by 10mg for another 3 days. During this period and up to 9 weeks afterwards HEV replication reactivated and the serum viral load increased to 2.3×10^4^ copies per mL.

Ribavirin treatment was complicated by anemia with a decline in hemoglobin concentration from 13.6 to 10.7g/dL, and thus an increase in dosing to 1200mg daily had to be postponed for 1 month. On this treatment dosing, he cleared the HEV infection in 1.5 months and treatment with ribavirin continued for an additional 3 months (i.e. total treatment duration: 800mg/day for 4.5 months and 1200mg/day for 4.5 months), and a sustained virological response (SVR) was achieved. A liver biopsy 1 year later revealed lack of fibrosis and only minor, non-significant inflammation (grade 0 to 1 according to Batts–Ludwig classification).

### Tracing source of infection

HE is a notifiable disease in Sweden, and possible routes of infection should be investigated. The patient lived and worked in Gothenburg and had not travelled outside Sweden during the last years. He reported no close contact with pigs or other animals, except for birds. No additional cases of HEV infection were diagnosed at the ward during his hospitalization. Sera from the heart donor was retrospectively investigated and found negative for both HEV RNA and anti-HEV IgG and IgM. During surgery and shortly thereafter, the patient received considerable amounts of blood-derived products including thrombocytes, clotting factors and plasma. The clotting factors were produced from a pharmaceutical company assuring that all blood products were tested and negative for HEV RNA in pooled samples. In total thrombocytes, erythrocytes and plasma from 17 different donors were administered to the patient. One year after the transplantation, all 17 donors were asked to provide a serum sample for serological investigation of HEV markers. Of the donors, 12 accepted, and among them, one had detectable anti-HEV IgG. This donor had anti-HEV IgG at a high titer, but had undetectable anti-HEV IgM and HEV RNA in serum. Despite repeated efforts, the remaining five donors did not provide serum samples.

**Fig. 2:**
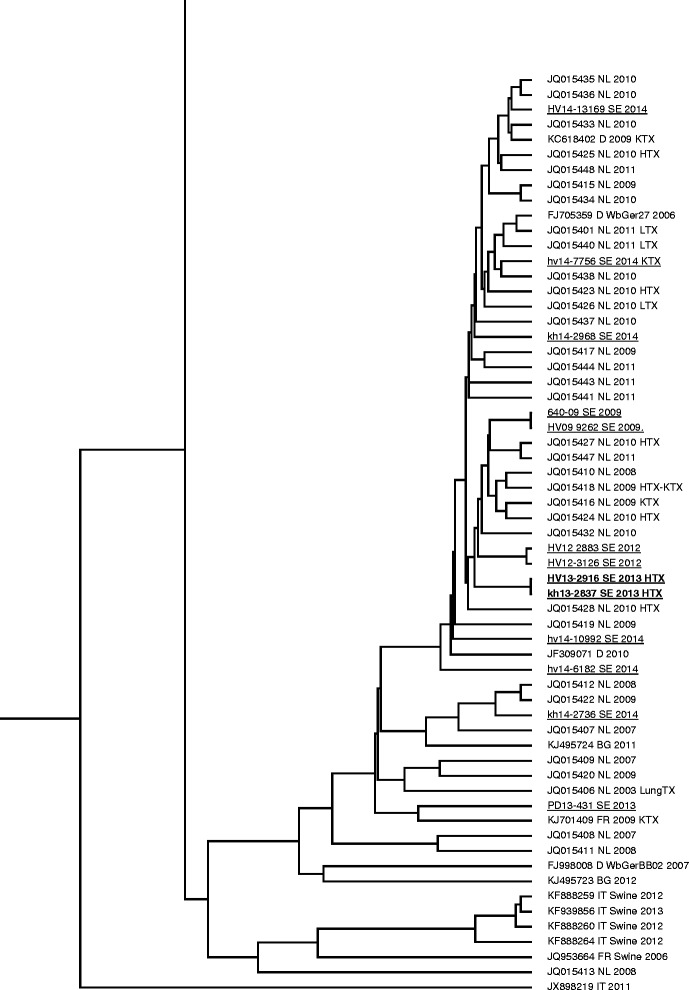
Phylogenetic tree based on 337 nucleotides of partial hepatitis E virus polymerase region of open reading frame 1 of 233 genotype 3 strains. The strains divide into two major groups designated 3I and 3II. The branch within 3II formed by strains circulating in Europe including the strain infecting the patient described in this study is enlarged. The accession numbers, origin and the derivation of the strains from a transplanted patient or other hosts such as swine or wild boar is given at each tip of the branches. *BG* Bulgaria, *D* Germany, *HTX* heart transplant patient, *IT* Italy, *KTX* kidney transplant patient, *LTX* liver transplant patient, *lungTX* lung transplant patient, *NL* The Netherlands, *SE* Sweden. Swedish patient strains are *underlined* and the strain infecting the patient in this case report is shown in *bold*

To obtain the gt and an indication of the origin of the infecting strain, the viral genome was amplified, sequenced and phylogenetically analyzed in the polymerase region of ORF1 as previously described [19]. A phylogenetic tree based on the sequences obtained and the corresponding genomic region in 120 HEV strains obtained from GenBank showed that the infecting virus from the transplanted patient was a HEV strain belonging to gt 3. This strain was found on a branch in the phylogenetic tree formed by a HEV strain from German wild boar and from individuals with clinical manifestation of HEV infection during the last 4 years in the Netherlands, Sweden, Germany, and Bulgaria (Fig. 2).

## Discussion

This study describes chronic HEV infection in a heart transplant recipient who probably was infected at the time of transplantation or during the postoperative hospitalization. HEV RNA was present 21 days or earlier after transplantation, indicating that the patient was infected at or in close proximity to the time of surgery. The infection was ongoing with presence of HEV RNA in serum several months before the patient had a peak in liver enzymes or a detectable antibody response. The HE was not found in a screening investigation, but in an acute investigation because of very high enzyme levels which peaked at 1100IU/mL and coincided with high viral levels. His alanine aminotransferase (ALT) levels were higher than usual for an immunocompromised patient, where values below 300IU/mL are expected, even if ALT values as high as up to 1506IU/mL have been shown in some of these patients [20, 21]. This observed delay in elevated liver enzymes and seroconversion is consistent with other studies showing that ALT elevations may occur before, simultaneously or after detection of hepatitis E viremia.

Anti-HEV IgM and IgG in sera has been reported detectable between 0 and 826 days after the occurrence of HEV RNA in sera from solid organ recipients, and even complete absence of seroconversion may occur [22, 23]. Since reduced immunosuppression, and if needed treatment with ribavirin, yields high cure rates [21], there ought to be a low threshold for PCR-based testing for HEV in patients with suppressed immunity.

The post-transplant dosing of tacrolimus was somewhat high, which may, in combination with the decrease of mycophenolate mofetil, have worsened the development of the infection. A recent study has shown that HEV replication *in vitro* increases when treated with high dose tacrolimus, and decreases when treated with mycophenolic acid [24]. Also, a significant association between the use of mycophenolate mofetil and clearance of infection has been noted [22]. Mycophenolate acid, like ribavirin, inhibits inosine monophosphate dehydrogenase. Tacrolimus-based immunosuppression reportedly is one of two independently associated risk factors for developing chronic infection among solid organ recipients [17]. Further studies are needed to investigate if treatment with mycophenolate mofetil may decrease the risk for developing chronic HEV infection in solid organ recipients.

The route of transmission could not be determined for the infection described in this study. Patient-to-patient transmission may have occurred as well as infection from contaminated food products ingested at the ward just prior to transplantation. However, none of the other patients treated on the same ward was diagnosed with hepatitis E. The first post-surgery serum sample available from the patient was obtained 3 weeks after transplantation and had detectable HEV RNA as the only marker of HE. This sampling was within or at the end of the incubation period for hepatitis E, which indicates that the patient became infected at or close to the time of the transplantation. The infection was not from the heart donor, who was negative for all hepatitis E markers, but one of the 17 blood donors had high anti-HEV IgG titer 1 year after donation and possibly could have been the source. This could not, however, be shown with certainty, since five of the donors could not be investigated for anti-HEV a year after surgery, and no stored samples from the blood donors were available from the time of transplantation. It is known that HEV RNA frequently is detected in blood donor samples and is found in up to 1 of 2200 blood donations, and that these blood donors often lack detectable anti-HEV IgG and/or IgM [25–29]. In addition, the patient received plasma-derived clotting factors produced by a major fractionator of Swedish plasma that performs HEV RNA testing on pooled plasma. It is unlikely that these factors may have infected the patient, but low concentrations of HEV RNA may fail to be detected in pooled samples tested for viral RNA, as has been shown by indirect evidence [30].

Phylogenetic analysis revealed that the infecting virus strain belonged to a clade formed by gt 3 strains in group 3I [19]. The strains forming this clade have been isolated in samples from patients with symptomatic HE in Europe during recent years, including Sweden where it has spread and caused clinical infections during the last years. This strain thus is endemic, consistent with the lack of travel history for the patient described in this study. Whether or not autochthonous hepatitis E caused by this strain is an emerging problem, or if it has been circulating for a longer period remains to be determined.

## Conclusions

Further studies are warranted to evaluate the extent of chronic hepatitis E among Swedish recipients of solid organs to minimize the risk of cirrhosis and fibrosis in this population, and to evaluate possible treatment regimens. Furthermore, it is important to determine whether there is a need for screening blood donors, particularly if blood products are to be given to immunosuppressed patients.

## Consent

Written informed consent was obtained from the patient for publication of this case report and accompanying images. A copy of the written consent is available for review by the Editor-in-Chief of this journal.
